# Supplementary material for: Brazilian fossils reveal homoplasy in the oldest mammalian jaw joint
Source: Nature. 2024 Sep 25;634(8033):381–8. doi: 10.1038/s41586-024-07971-3 (PMC11464377; doi:10.1038/s41586-024-07971-3)
Supplement: Supplementary file 1 — Supplementary Notes, Supplementary Figs. 1 and 2 and Supplementary Tables 1 and 2. [file 41586_2024_7971_MOESM1_ESM.pdf]

nature portfolio

https://doi.org/10.1038/s41586-024-07971-3

Supplementary information

Brazilian fossils reveal homoplasy in the oldest mammalian jaw joint

In the format provided by the
authors and unedited

Supplementary Information for "Brazilian fossils reveal homoplasy in the oldest mammalian jaw joint"

James R. G. Rawson, Agustín G. Martinelli, Pamela G. Gill, Marina B. Soares, Cesar L. Schultz, Emily J. Rayfield

1. Supplementary Information – List of supplementary files provided, available at the University of Bristol data repository, data.bris, at https://doi.org/10.5523/bris.2ie8neiry701e23iayx9gdsytv.

MPG video files containing animations of surface models generated from segmented micro-CT data gathered in this study. Separate colours in the segmentation denote the cranium (yellow), coronoid (dark blue), dentary (orange), postdentary bones (blue), quadrate (green), quadratojugal (purple), splenial (pink), and stapes (cyan).

Brasilodon **UFRGS-PV-628-T animation.mpg:** Animation of surface model of the skull and left jaw of UFRGS-PV-628-T (*Brasilodon*), showing two full yaw and two full roll rotations. The right jaw, not shown here, was removed in preparation.

Brasilodon **UFRGS-PV-628-T jaw animation.mpg:** Animation of surface model of the left jaw only of UFRGS-PV-628-T (*Brasilodon*), showing two full yaw and two full roll rotations. This video shows the full anatomy of the jaw, including the preserved coronoid bone.

Brasilodon **UFRGS-PV-929-T animation.mpg:** Animation of surface model of the skull and jaws of UFRGS-PV-929-T (*Brasilodon*), showing two full yaw and one full roll rotations.

Brasilodon **UFRGS-PV-1030-T animation.mpg:** Animation of surface model of the skull and jaws of UFRGS-PV-1030-T (*Brasilodon*), showing two full yaw and two full roll rotations.

Brasilodon **UFRGS-PV-1043-T jaw animation.mpg:** Animation of surface model of the jaws of UFRGS-PV-1043-T (*Brasilodon*), showing two full yaw and two full roll rotations. These jaws were scanned separately from the skull. Some isolated postcranial elements present in the block, likely limb and rib material, are not shown in this animation. The left jaw of this specimen was used as the base of the jaw reconstruction shown in Extended Data Figure 1.

Brasilodon **UFRGS-PV-1043-T skull animation.mpg:** Animation of surface model of the complete skull of UFRGS-PV-1043-T (*Brasilodon*), showing two full yaw and two full roll rotations. A largely complete right stapes is preserved displaced within the skull, and the right quadrate is preserved in situ.

Riograndia **UFRGS-PV-596-T animation.mpg:** Animation of surface model of the complete skull of UFRGS-PV-596-T (*Riograndia*), showing two full yaw and two full roll rotations. This specimen preserves a largely compete skull and right jaw, including the jaw articulation in good detail. A small quadratojugal is also visible in this

specimen. The right jaw of this specimen was used as the starting point for the complete jaw reconstruction shown in Extended Data Figure 2.

Riograndia **UFRGS-PV-833-T animation.mpg:** Animation of surface model of the left dentary and postdentaries of UFRGS-PV-833-T (*Riograndia*), showing two full yaw and two full roll rotations. The postdentary bones and teeth of this specimen were used to assemble the complete jaw reconstruction shown in Extended Data Figure 2.

Riograndia **UFRGS-PV-1319-T animation.mpg:** Animation of surface model of the right dentary and fragmentary postdentary of UFRGS-PV-1319-T (*Riograndia*), showing two full yaw and two full roll rotations.

Riograndia **MPDC-1B1 animation.mpg:** Animation of surface model of the left dentary of MPDC-1B1 (*Riograndia*), showing two full yaw and two full roll rotations.

STL files containing surface models generated from segmented micro-CT data gathered in this study. Models were exported directly from surfaces generated in Avizo 3D 2021.1 (*Brasilodon*, *Riograndia*) or Dragonfly 2022.2 (*Oligokyphus*).

Brasilodon **UFRGS-PV-628-T.stl:** Surface model of the skull and left jaw of UFRGS-PV-628-T (*Brasilodon*) generated from segmented CT data. This specimen also preserves a right quadrate.

Brasilodon **UFRGS-PV-929-T.stl:** Surface model of the skull and jaws of UFRGS-PV-929-T (*Brasilodon*) generated from segmented CT data. This specimen preserves both jaws fairly completely, with a right quadrate and quadratojugal preserved in situ.

Brasilodon **UFRGS-PV-1030-T.stl:** Surface model of the skull and left jaw of UFRGS-PV-1030-T (*Brasilodon*) generated from segmented CT data. This specimen preserves a quadrate in articulation with the postdentary bones on the right side.

Brasilodon **UFRGS-PV-1043-T.stl:** Surface model of the left and right dentary and disarticulated postdentary bones of UFRGS-PV-1043-T (*Brasilodon*) generated from segmented CT data. The skull of UFRGS-PV-1043-T is preserved separately and so is included as a separate STL file (see below).

Brasilodon **UFRGS-PV-1043-T skull.stl:** Surface model of the complete skull of UFRGS-PV-1043-T (*Brasilodon*) generated from segmented CT data, scanned separately from the lower jaws. This specimen also preserves a slightly distorted right stapes displaced to the centre of the skull.

Brasilodon **Jaw Reconstruction.stl:** Surface model of the complete jaw reconstruction of *Brasilodon quadrangularis* created in this study. The process of reconstructing this jaw is shown in Extended Data Figure 1. The left jaw of UFRGS-PV-1043-T was used as the basis for the reconstruction, and small cracks and breakages were repaired using the segmentation editor in Avizo 3D 2021.1. The left splenial and postdentaries were then moved into hypothesised life position based on data from other specimens. Right postdentary elements preserved in UFRGS-PV-1043-T were then mirrored over (Extended Data Figure 1c). The coronoid was segmented from UFRGS-PV-628-T (Fig. 2g) and scaled using equivalent measurements of the jaws and skull. The remaining

missing portions of the dentary and postdentaries were reconstructed in Blender 3.4 based on data from other specimens where the dentary and postdentaries were more complete.

Oligokyphus **NHMUK-PV-R7119.stl:** Surface model of NHMUK-PV-R7119 (*Oligokyphus*), a largely complete right dentary, generated from segmented CT data.

Oligokyphus **NHMUK-PV-R7121.stl:** Surface model NHMUK-PV-R7121 (*Oligokyphus*), an anterior right dentary, generated from segmented CT data.

Oligokyphus **NHMUK-PV-R7189.stl:** Surface model of NHMUK-PV-R7189 (*Oligokyphus*), the posterior of a left postdentary complex, generated from segmented CT data. This specimen likely contains elements of the articular, surangular and prearticular.

Oligokyphus **NHMUK-PV-R7190.stl:** Surface model of NHMUK-PV-R7190 (*Oligokyphus*), the posterior of a right postdentary complex, generated from segmented CT data. This specimen likely contains elements of the articular, surangular and prearticular.

Oligokyphus **NHMUK-PV-R7196.stl:** Surface model of NHMUK-PV-R7196 (*Oligokyphus*), a right quadrate, generated from segmented CT data.

Oligokyphus **NHMUK-PV-R7204.stl:** Surface model of NHMUK-PV-R7204 (*Oligokyphus*), a right dentary, generated from segmented CT data. This specimen is made up of an anterior and posterior portion.

Oligokyphus **NHMUK-PV-R7373.stl:** Surface model of NHMUK-PV-R7373 (*Oligokyphus*), an anterior portion of the left dentary, generated from segmented CT data.

Riograndia **UFRGS-PV-596-T.stl:** Surface model of the skull and right jaw of UFRGS-PV-596-T (*Riograndia*) generated from segmented CT data. This specimen preserves the right jaw joint region in good detail, including an in-situ quadrate and quadratojugal. The postdentary bones on the right jaw are also well-preserved.

Riograndia **UFRGS-PV-833-T.stl:** Surface model of the left jaw of UFRGS-PV-833-T (*Riograndia*) generated from segmented CT data. The left postdentary complex is well-preserved in this specimen. The skull of UFRGS-PV-833-T is also present in the same block but was not segmented in this study.

Riograndia **UFRGS-PV-1319-T.stl:** Surface model of the right dentary and fragmentary postdentary of UFRGS-PV-833-T (*Riograndia*) generated from segmented CT data.

Riograndia **MPDC-1B1.stl:** Surface model of the left dentary of MPDC-1B1 (*Riograndia*) generated from segmented CT data.

Riograndia **Jaw reconstruction.stl:** Surface model of the complete jaw reconstruction of *Riograndia guaibensis* created in this study. The process of reconstructing this jaw is shown in Extended Data Figure 2. The right jaw of UFRGS-PV-596-T was used as the basis for the reconstruction, and small cracks and breakages were repaired using the

segmentation editor in Avizo 3D 2021.1 (Extended Data Figure 2a). The left postdentaries and broken sections of the posterior dental were then moved into hypothesised life position (Extended Data Figure 2b). The more complete left postdentary bones of UFRGS-PV-833-T (Extended Data Figure 2c) and the left coronoid of UFRGS-PV-624-T (Extended Data Figure 2d-e) were then mirrored over and scaled using equivalent measurements of the dentary and, in the case of UFRGS-PV-833-T, the skull. The remaining missing portions of the dentary and coronoid were reconstructed in Blender 3.4.

Files containing the updated character matrix used to carry out the phylogenetic analysis used in this study. Matrix is provided in both TNT and Mesquite compatible formats.

Phylogenetic_matrix_nexus.nex: Phylogenetic character matrix in nexus format.

Phylogenetic_matrix_nexus.tnt: Phylogenetic character matrix in TNT format

2. Supplementary Text – Descriptions of the lower jaws of *Brasilodon quadrangularis* and *Riograndia guaibensis*

Brasilodon quadrangularis

Our µCT scans allow us to supplement and verify previous descriptions of the lower jaw of *Brasilodon* [6-8,87,93]. The dentary of *Brasilodon* is slender and elongate, with a slight upward curvature at the anterior end where a diastema develops between the canine and postcanines of larger specimens (e.g. UFRGS-PV-628-T, UFRGS-PV-1043-T). The coronoid process angles posteriorly and becomes more pronounced in larger specimens, and there is no suggestion of a distinct angular process. The lateral and medial ridges of the dentary form the articular process, though this process is less developed than previous descriptions suggested [6-8]. As discussed in the main text, we do not find evidence of a dentary-squamosal contact in *Brasilodon*. The postdentary trough, which in cynodonts holds the postdentary bones, is narrow, straight, slightly ventrally inclined and terminates roughly in line with the posterior margin of the tooth row. The Meckelian groove runs longitudinally and terminates in the anterior part of the unfused symphysis, never straying on to the ventral surface of the dentary. The coronoid bone has been identified previously in UFRGS-PV-603-T [6], but µCT data also reveals this bone is present on both sides of UFRGS-PV-929-T, UFRGS-PV-1043-T and on the left jaw of UFRGS-PV-628-T. The coronoid is rhomboid in medial profile and attaches to the medial surface of the dentary just posterior to the postcanines. In UFRGS-PV-628-T, the coronoid preserves a stout posterior projection resembling that of other cynodonts [57] and

Morganucodon [28]. UFRGS-PV-1043-T preserves two partial splenials obscured by the matrix in the physical specimen. The profile of the splenial matches an indentation on the anteromedial portion of the dentary, demonstrating that the complete splenial would have covered the Meckelian groove posterior to the mandibular symphysis. The splenial is rounded anteriorly and narrows posteriorly in medial view. It is mediolaterally flat and possesses a shallow anteroposterior groove on its lateral surface that would presumably have accepted Meckel's cartilage in life. UFRGS-PV-1043-T also possesses a displaced right stapes described in detail by Rodriguez et al. (2013)[39], and μCT scans corroborate the noted similarity to other derived probainognathians.

The postdentaries of *Brasilodon* generally resemble those of probainognathian cynodonts like *Probainognathus* and *Pachygenelus* in size and shape [3,5,48] more closely than they do the reduced postdentaries of mammaliaforms. The articular, prearticular and surangular are fused in all specimens apart from in the small UFRGS-PV-1030-T, where a distinct suture line separating the surangular is visible in the μCT scans. The articulated jaw joint preserved in UFRGS-PV-1030-T and UFRGS-PV-929-T suggests that the surangular did not articulate with the quadrate. The surangular runs dorsally above the prearticular and articular and is barred from contacting the squamosal by the lateral ridge of the dentary. The anterior margin of the surangular terminates posterior to that of the prearticular, which reaches the anterior margin of the postdentary trough. The retroarticular process is often poorly preserved but is most prominent in UFRGS-PV-1043-T, where it projects anteroventrally from main body of the articular. The angular is not fused to the other postdentary bones and is preserved separately in several specimens, but would have run parallel to the prearticular, articular and surangular in life. The reflected lamina of the angular juts out ventrally from the rest of the angular posterior of the ventral margin of the postdentary trough. It is small and often poorly preserved, though its anatomy can largely be reconstructed using specimens UFRGS-PV-628-T, UFRGS-PV-1030-T and UFRGS-PV-1043-T.

Riograndia guaibensis

The lower jaw of *Riograndia* has been described thoroughly in previous works [9,94], but we are able to add a few details made available by our μCT scans. The general shape of the dentary in *Riograndia* is markedly different to that of *Brasilodon* and more resembles tritheledontids

like *Pachygenelus* [4], though *Riograndia* is even more robust and dorsoventrally deep in construction. The dentition has been thoroughly described [9,94] and no additional information was recovered from the μCT scans. The coronoid process is high, rounded, and inclined posteriorly. The unfused symphysis is particularly large and inclines anteriorly. The postdentary trough is dorsoventrally tall to accommodate the large postdentary bones, with a dorsally concave roof formed by the lateral and medial ridges of the dentary. These ridges terminate in a large articular process that prevents any articulation between the surangular and the squamosal and forms the dentary-squamosal contact, described more thoroughly in the main text. The Meckelian groove runs longitudinally along the jaw and onto the symphysis, never touching the ventral surface of the dentary. Unlike *Brasilodon*, *Riograndia* bears a distinct angular process ventral to the postdentary trough. There is no evidence of a preserved splenial in any of the specimens. The μCT scans did not support the presence of a coronoid bone in UFRGS-PV-596-T [9], but it is present in UFRGS-PV-624-T. The coronoid is small in comparison with that of less derived probainognathians and attaches close to the anterior margin of the medial side of the coronoid process, dorsal to the penultimate postcanine.

The postdentaries of *Riograndia* were described in Soares et al. (2011)[9] in good detail, but μCT scans permit observation of some additional anatomy previously obscured by matrix. Postdentary bones were found preserved in UFRGS-PV-596-T, UFRGS-PV-624-T, UFRGS-PV-833-T and UFRGS-PV-1319-T and are much more robust than those found in *Brasilodon*, resembling those of *Pachygenelus* and other more basal probainognathians [4,5,57]. The angular, prearticular and articular are fused in all specimens, though the μCT scans do preserve visible margins between the bones in some slices of UFRGS-PV-624-T. This specimen also preserves the anterior most portions of the postdentary bones that are obscured by matrix in the physical fossils, though they are quite fragmented. The anterior portion of the surangular runs dorsally to the fused prearticular and angular, with both elements tapering at the anterior margin of the postdentary trough to meet at the posterior margin of the Meckelian groove. The remaining anatomy corroborates the findings of Soares et al. (2011)[9], including the posteriorly projected retroarticular process and the lack of a preserved reflected lamina on the angular. The retroarticular process is largely made up of the articular, though there is some contribution from the angular on the ventrolateral portion.

3. Supplementary Text – Jaw articulation in *Oligokyphus major*

An obvious comparison for the revised anatomy of *Brasilodon* are the tritylodontids, a group of probainognathians that are often positioned adjacent to *Brasilodon* in phylogenetic analyses (Fig. 1) [34,38,56] and which have also lost the surangular-squamosal contact [4,49]. Our μCT scans of the Late Triassic-Early Jurassic tritylodontid *Oligokyphus* (Extended Data Figure 3) corroborate previous descriptions of the jaw joint region [50,95], showing that the small quadrate-articular joint was the only point of articulation in this early diverging tritylodontid, much like *Brasilodon*. The articular process of the dentary of tritylodontids is less developed than in *Brasilodon*, and we found no osteological evidence of a synovial joint between the squamosal and the dentary in *Oligokyphus*. Nor did we find evidence of a ligamentous connection between the jugal and dentary, as has been suggested in previous descriptions [4]. Fourie (1968)[96] advocated for the presence of a dentary squamosal joint in the more derived tritylodontid *Tritylodon* based on description of a single large specimen (at the time referred to as *Tritylodontoideus maximus*) preserved as a positive cast. However, this specimen was heavily distorted by taphonomic processes, and we are sceptical of this interpretation in lieu of better-preserved individuals being thoroughly described. We therefore take the position adopted by others (e.g. [97]) that *Tritylodon* was not in possession of a dentary-squamosal joint. Additionally, the derived position of *Tritylodon* within tritylodontids would suggest that, were Fourie's interpretation correct, the presence of a dentary-squamosal joint would be a derived autapomorphy of this taxon and therefore unlikely to affect our conclusion that the dentary-squamosal contact evolved separately in ictidosaurs and Mammaliaformes.

The delicate postdentary bones possess no surangular boss to articulate with the squamosal, confirming that the surangular-squamosal articulation is also absent. Tritylodontids therefore lack any reinforcement of the quadrate-articular joint and the small size of this articulation in *Oligokyphus* suggests that it could not have been under any significant stress, which would likely have also been the case in *Brasilodon*. Other features of the jaw joint region, shown in our μCT scans of *Oligokyphus* matched those of previous descriptions [50,95].

4. Supplementary Text– Phylogenetic analysis methods

The character list and data matrix are based on previous studies focused on non-mammaliaform cynodonts. The character list follows the original proposal of Liu and Olsen (2010) [38] which is mostly based on Hopson and Kitching (2001) [61], plus data obtained from Hopson and Barghusen (1986) [58], Rowe (1998) [19], Wible (1991) [63], Wible and Hopson (1993) [64], Lucas and Luo (1993) [98], Luo (1994) [60], Luo and Crompton (1994) [47], Martínez et al. (1996) [99], Luo et al. (2001) [100], Bonaparte et al. (2003, 2005) [6,7], Sidor and Smith (2004) [101]; Martinelli et al. (2005) [67], Abdala (2007) [36], Martinelli and Rougier (2007) [37], among others. The data-matrix of Liu and Olsen (2010) [38] was subsequently used as base for most but not all (e.g., Ruta et al. 2013 [56]; Gaetano et al., 2021 [102]) subsequent studies, including successive character and terminal operational units additions and character and character-states modifications by Soares et al. (2014) [33], Martinelli et al. (2016 [34], 2017 [103], 2024 [104]), Wallace et al. (2019) [42], Kerber et al. (2022 [45], 2024 [105]), Benoit et al. (2022) [43], Stefanello et al. (2023) [106], among others. We made the following modifications to the data matrix of Martinelli et al. (2024) [104]:

1) Character 7. *Irajatherium* changes from ? to 1 (Maxilla participates in the border of subtemporal fenestra).
2) Character 20. *Irajatherium* changes from 1 to 0 (Zygomatic arch dorsal extent, below middle of the orbit).
3) Character 42. *Riograndia* changes from 0 to 1 (Posterior portion of secondary palate almost at the level of the tip of the postcanine upper teeth, forming a deep groove between the hard palate and the tooth row).
4) Character 66. *Riograndia* changes from 0 to 1 (Paroccipital process in the base of the posttemporal fossa, present).
5) Character 78. *Riograndia* changes from ? to 1 (Articulation of the quadrate with the squamosal, covered dorsally by the squamosal).
6) Character 80. *Riograndia* changes from ? to 2 (incipient dentary/squamosal craniomandibular articulation). *Brasilodon* changes from 2 to 0 (quadrate/articular craniomandibular articulation). *Trucidocynodon* changes from 0 to 1 (based on holotype UFRGS-PV-1070-T) (main quadrate/articular craniomandibular articulation, secondary surangular/squamosal).
7) Character 82. Shape of squamosal articulation surface for mandible: small and medially or anteromedially facing facet (0); wide, ventrally directed glenoid cavity

(1). This character is ch. 81 in Wallace et al. (2019) [42], who modified the original character definition by deleting its stage 0 [absent (0)]. However, they scored erroneously almost all taxa as having a squamosal articulation with mandible and it was kept in successive works after it. We changed *Procynosuchus*, *Galesaurus*, *Thrinaxodon*, *Platycraniellus* from 0 to (-) inapplicable. *Cynognathus*, *Diademodon*, *Trirachodon*, *Sinognathus*, *Exaeretodon*, *Massetognathus*, *Luangwa*, *Probainognathus*, *Pachygenelus* and *Ecteninion* from 1 to 0. *Chiniquodon* and *Pseudotherium* from 1 to inapplicable. *Lumkuia* from inapplicable to 0. *Brasilodon* from ? to inapplicable. All tritylodontids from 0 to inapplicable.

8) Character 99. *Riograndia* changes from 0 to 1 (Distinct upper incisor/canine diastema, absent)
9) The operational unit *Riojanodon nenoi*, *Cromptodon mamiferoides* and CRILAR-Pv 567 introduced in Martinelli et al. (2024) [104] were deleted considering their fragmentary nature and are not scope of this contribution.

The data matrix is provided in separate files in Mesquite and TNT formats.

5. Supplementary Text – List of Characters

Rostrum

1. Premaxillary extranasal process: absent or with very little exposure (0); large but not contacting nasal (1); contacting nasal (2).
2. Septomaxilla facial process: long, far beyond the posterior border of the external nares (0); short, almost limited in the external nares (1).
3. Snout in relation to the temporal region (to the posterior border, not the parietal crest): longer (0); subequal (1); shorter (2).
4. Paracanine fossa in relation to the upper canine: anteromedial (0), medial or posteromedial (1), anterior (2), absent (3).
5. Premaxilla forms posterior border of the incisive foramen: absent (0), present (1).
6. Maxillary platform lateral to the teeth series: absent (0); present (1).
7. Maxilla: excluded from (0), or participates in (1) border of subtemporal fenestra.

Skull roof

8. Profile of skull roof: nearly flat (0); remarkably concave (the parietal crest is higher than the extension of anterior surface) (1); convex (the parietal crest is lower than the extension of anterior surface) (2).

9. Parietal foramen: present (0); absent (1).

10. Interparietal (postparietal) in adult: separate bone in adult (0); fused with other bones (1).

11. Lateral expansion of braincase in parietal region: absent (0); well-developed (1).

12. Parietal crest posteriorly extending close to or reach the posteriomost position of the occipital crest: absent (0); present (1). [LO12]

Orbital region

13. Prefrontal: present (0); absent (1).

14. Postorbital bar and postorbital: present (0); postorbital present but not forming postorbital bar (1); both absent (2).

15. Palatine: do not meet the frontal (0); meets frontal but two elements without significant contribution to medial orbit wall (1); meets frontal and two elements with significant contribution to medial orbit wall (2).

Zygomatic arch

16. Sphenopalatine foramen: absent (0); present (1).

17. Zygomatic arch dorsoventral height relative to skull length: moderately deep (10~18%) (0); very deep (>18%) (1); slender (2) (<10%).

18. The anteroventral corner of the zygomatic arch: lie at the same level as (0); or remarkably higher than (1) the postcanine line.

19. Infraorbital process: absent (0); suborbital angulation between maxilla and jugal (1); descending process of the jugal (2).

20. Zygomatic arch dorsal extent: below middle of orbit (0); above middle of orbit but still level within orbit (1); beyond the upper border of the orbit (2).

21. Posterior extension of jugal along zygomatic arch: extending back near quadratojugal notch of squamosal (0), extending back near squamosal glenoid (1), reduced and receding from glenoid (2).

22. The posteroventral process of jugal: low (0); high, forming more than half height of zygomatic arch (1).

23. The width of temporal fossa: reach greatest near middle (0); same throughout or little change (1); strongly increase backward, the posterior width much bigger than the anterior width (2).

24. Squamosal groove for external auditory meatus: without or with an incipient depression (0); deep (1).

25. Posterior extension of the squamosal dorsal to the squamosal sulcus in zygomatic arch: incipient (0); well developed (1).

26. The notch separating lambdoidal crest from zygomatic arch: shallow (0); deep, “V”-shape (1).

Palatal complex

27. Palatine: excluded from subtemporal border of orbit (0); participates in subtemporal border by displacing pterygoid posteriorly (1).

28. Vomer exposure in incisive foramen (at anterior ends of maxillae on palate): present (0); absent (1).

29. Vomer: with (0) or without (1) vertical septum extending posterior to level of secondary palate.

30. Ectopterygoid: does not contact maxilla (0); contacts maxilla (1); absent (2).

31. Interpterygoid vacuity in adults between pterygoid flanges: present (0); absent (1).

32. Secondary palatal plate on maxilla reaches midline: absent (0); present (1).

33. Secondary palatal plate on palatine reaches midline: absent (0); present (1).

34. Posterior extent of osseous secondary palate: far from (0), close to or beyond (1) rear upper postcanine row.

35. The posterior end of secondary osseous palate relative to anterior border of orbit: anterior (0); about equal level (1); posterior (2).

36. Osseous palate extension: 45% of skull length or less (0); more than 45% of skull length (1).

37. Length of palatine relative to maxilla in secondary palate: (0) shorter; (1) about equal; (2) longer.

38. Middle of pterygoid: smooth (0); a boss (1); a distinct median crest (2).

39. The nasopharyngeal roof posterior to the transverse process of pterygoid: narrow, deep, ventrally forms a keel (0); wide, flat, the narrowest place greater than half the width of the transverse process (1).

40. Quadrate ramus of pterygoid: present (0); absent (1).

41. Quadrate articulation with quadrate ramus of epipterygoid: absent (0); present (1).
42. Posterior portion of secondary palate almost at the level of the tip of the postcanine upper teeth, forming a deep groove between the hard palate and the tooth row: (0) absent; (1) present.

Basicranium, and lateral wall of the braincase
43. Frontal-epipterygoid contact: present (0), absent (1).
44. Epipterygoid ascending process at level of trigeminal foramen: greatly expanded (0); moderately expanded (1).
45. The anterior part of the basisphenoid: narrow (0); wide, and the width greater than half the width of the transverse process (1).
46. Parasphenoid ala: at the same level as the basicranium (0); ventrally expanded below the basicranium (1).
47. Basisphenoid wing (parasphenoid ala): long, border the fenestra vestibuli (0); slightly reduced and excluded from oval window, overlap the entire prootic cochlear housing (1); greater reduced and overlapping a part of the pars cochlearis (cochlear housing) (2); basisphenoid does not overlap the petrosal pars cochlearis (3).
48. Overlap of the basioccipital to the pars cochlearis: entire cochlear housing (0); the medial side of the promontorium (1); no overlapping (2).
49. Internal carotid foramina in basisphenoid: present (0); absent (1).
50. Prootic and opisthotic: separated (0); fused at early ontogenetic stage to form petrosal (=periotic) (1).
51. Promontorium (Pars cochlearis of petrosal): absent (0); present (1).
52. Internal auditory meatus: open (0); walled (1).
53. The trigeminal ganglion (semilular ganglion): open ventrally (0); partial prootic floor (1); complete prootic floor (2).
54. Lateral trough floor anterior to the tympanic aperture of the prootic canal and/or the primary facial foramen: absent (0); present (1).
55. Vascular foramen in the posterior part of the lateral flange (Foramen "X" of Rougier et al., 1992: 205): absent (0); present (1).
56. Foramen and passage of prootic sinus on lateral trough: absent (0); present (1).
57. Route of the venous drainage exiting from the back of the cavum epiptericum: only lateral flange vascular groove (0); absent (1); lateral flange vascular canal present (foramina on lateral surface) (2).

58. Maxillary and mandibular branch (V2+3) of the trigeminal nerve exit: via single foramen between prootic and epipterygoid (0); via two foramina between prootic and epipterygoid (1); via separate foramina, some enclosed by anterior lamina of prootic (petrosal) (2).

59. Pterygoparoccipital foramen: squamosal does not contribute to enclosure of foramen (0); squamosal contributes to enclosure of foramen (1); open (2).

60. Vertical component of lateral flange of prootic (“L-shaped” and forming a vertical wall to pterygoparoccipital foramen): absent (0); present (1).

61. Anterior part of paroccipital process: the lateral aspect covered by the squamosal (0); exposed due to dorsally withdrawn of the squamosal (1).

62. Hyoid (stapedial) muscle fossa on the paroccipital process: absent (0); present (1).

63. Paroccipital process: undifferentiated (0); differentiated (1).

64. Fenestra rotunda separation from jugular foramen: (0) confluent; (1) partially separated by finger-like projection from postero-lateral wall of jugular foramen; (2) completely separated.

Occipital region

65. Articulation of the paroccipital process with the quadrate: absent (0); present (1).

66. Paroccipital process in the base of the posttemporal fossa: absent (0); present (1).

67. Tabular: present (0), absent (1).

68. The relationship of hypoglossal foramen (condylar foramen) with the jugular foramen: confluent or sharing a depression (0); at least one foramen completely separated from the jugular foramen (1).

69. Shape of the occipital condyles (in lateral view): bulbous (0); ovoid to cylindrical (1).

Craniomandibular joint

70. Rotation of dorsal plate relative to trochlear axis on quadrate: less than 10 degree (0); about 45 degrees (1); around 90 degrees (2); parallel to trochlear axis (3).

71. Curvature of the contact facet on the posterior side of the dorsal plate of quadrate: flat or convex (0); concave (1).

72. Size of the lateral trochlear condyle relative to the medial trochlear condyle on quadrate: the lateral condyle larger than the medial condyle (0); the medial condyle equal or larger than the lateral condyle (1).

73. Shape of the trochlear of quadrate: cylindrical (0); trough-shaped (1).

74. Lateral margin of the dorsal plate of quadrate: straight (0); flaring posteriorly (1); flaring and rotated posteromedially (2).

75. Medial margin of the dorsal plate of quadrate: straight (0); flaring anteriorly (1); flaring and rotated anterolaterally (2).

76. Dorsal margin of dorsal plate of quadrate: retains pointed angle (0); has rounded margin (1).

77. Lateral notch and neck of quadrate (separation of the lateral margin of the contact facet from the trochlear): the lateral notch is absent or poorly developed (0); lateral notch developed, separating the lateral margin of the contact facet from the lateral end of the trochlear (1); lateral notch is broader and separation of the lateral margin of contact facet for the trochlear is wider, the lateral margin is shifted medially (2); development of the neck with raise the contact facet away from the trochlear (3).

78. Articulation of the quadrate with the squamosal: via concave recess in the squamosal (0); covered dorsally by the squamosal (1); little or no contact with the squamosal (2).

79. Articulation of the quadrate with the stapes: via a broad recess on the medial margin and the median end of the trochlear (0); the stapedial contact restricted to the medial end of the trochlear (1); via a projection from the medial margin of the dorsal plate (2); via a medial vertical ridge in the neck (3); via a projection from the neck of the quadrate (4).

80. Craniomandibular articulation; quadrate/articular (0); main quadrate/articular, secondary surangular/squamosal (1); incipient dentary/squamosal (2); main dentary/squamosal (3).

81. Craniomandibular articulation: lies around the same height (0), much lower (1) or remarkably higher (2) than the postcanine line.

82. Shape of squamosal articulation surface for mandible: small and medially or anteromedially facing facet (0); wide, ventrally directed glenoid cavity (1).

Mandible

83. Dentary symphysis: unfused (0); fused (1).

84. Lateral ridge of the dentary: absent (0); incipient (1); moderately developed (2); strongly projected (3).

85. Angle of the dentary: close to the position of postorbital bar (0); close to the jaw joint (1).

86. Position of dentary-surangular dorsal contact relative to postorbital bar and jaw joint: around midway (0); closer to jaw joint (1).

87. Mediolateral thickening of the anterior margin of the coronoid process: absent (0); present (1).

88. Splenial: large and deep, reaches ventral border of the dentary (0); reduced to thin splint covering dentary groove (1).

89. Postdentary bones: large, with tall surangular (0); angular, surangular, and prearticular reduced in height and lying in dentary groove (1); further reduced to single gracile rod in postdentary trough (2).

90. Reflected lamina of angular posterior extent relative to distance from angle of dentary to jaw joint: greater than 1/2 the distance (0); less than l/2 the distance (1).

91. Reflected lamina of angular shape: spoon-shaped plate with slight depressions (0); hook-like lamina (1); reduced to thin process (2).

92. Mandibular movement during occlusion: orthal movement during power stroke (0); posteriorly directed power stroke (1); moderate rotation along the longitudinal axis in power stroke (2).

Dentition

93. Postcanine occlusion: lack consistent contact relationship (0); bilateral, interdigitating occlusion between multiple cusps (1); precise unilateral occlusion (2).

94. Relationships of wear facet to main cusp: wear facet absent (0); simple longitudinal facet on crown (1); main cusp bears two distinct facets (2); multiple cusps with each cusp bearing one or two transverse and crescentic facets (3); wear facet on lingual platform (4).

95. Upper incisors number: five or more (0); four (1); three or less (2).

96. Lower incisor number: four or more (0); three (1); two or less (2).

97. Incisor size: all of similar size (0); some incisors large (1).

98. Incisor cutting margins: smoothly ridged (0); serrated (1); denticulated (2).

99. Distinct upper incisor/canine diastema: present (0); absent (1).

100. Upper canine: large (0); reduced in size (<10% of skull length) (1); absent (2).

101. Lower canine: large (0); reduced in size (1); absent (2).

102. Canine serrations: absent (0); present (1).

103. Upper postcanine morphology: sectorial without or with incipient cingulum broadening the crown (0); sectorial with a well-developed lingual cingulum (1); bucco-lingually expanded, with large lingual cusp/cusps (2); sectorial with lingual platform (i.e., twice buccolingually broad than a complete lingual cingulum) (3).

104. Anterior most one-cusped tooth: present till adult (0); present only in juvenile (1); absent (2).

105. Posterior most gomphodont postcanine(s) in adults: absent (0); absent in juvenile but present in adult (1); present from juvenile (2).

106. Posterior postcanines with strongly curved main cusp: absent (0); present (1).

107. Upper postcanine roots: single (0); constricted root, with longitudinal groove (1); divided into two longitudinal aligned roots (2); multiple roots (more than two) (3).

108. Lower postcanine roots: single (0); constricted root, with longitudinal groove (1); divided in two roots (2); with multiple roots (more than two) (3).

109. Buccal (external) cingulum on sectorial upper postcanines: absent (0); present (1).

110. Number of upper cusps in transverse row: one (0); two (1); three or more (2).

111. Position of upper transverse cusp row on crown: midcrown (almost to posterior margin) (0); on anterior half of crown (1); at posterior margin (no posterior cingulum) (2).

112. Central cusp of upper transverse row: absent (0), midway between buccal and lingual cusps (1); closer to lingual cusp (2).

113. Arrangement of main cups of upper postcanines: in single longitudinal row (0); multiple cusps in multiple rows (1).

114. Interlocking of lower postcanines: absent (0); distal cuspule ‘d’ of anterior molar fits into embayment between cusp ‘b’ and cusp ‘e’ of the succeeding molar (1).

115. Number of lower cusps in transverse row: two (0), three or more (1).

116. Lingual cingulum on lower postcanine: present, not broader than the sectorial main labial cusps (0); vestigial or absent (1); present and forming a lingual platform, broader than the sectorial labial portion of the crown (2).

117. Lower posterior basin: absent (0); present (1). .

118. Axis of posterior part of maxillary tooth row: directed lateral to subtemporal fossa (0); directed toward centre of fossa (1); directed toward medial rim of the fossa and diverged (2); directed toward medial rim of the fossa and parallel (3).

119. Upper tooth series posterior extension: below the orbit and anterior to the subtemporal fenestra (0); anterior to the orbit (1); behind the anterior border of the subtemporal fenestra (2).

120. Postcanine replacement pattern: alternating (0); delayed (1); at most single replacement for one position (2); sequential addition of postcanines, no replacement (3).

121. Morphology of upper middle postcanines for taxa with sectorial teeth: symmetrical postcanines with main cusp A and small mesial and distal accessory cusps (0); mesiodistally asymmetrical postcanines with main cusp A and accessory mesial (usually one) and distal ones vertically oriented (1); mesiodistally asymmetrical postcanines with

main cusp A and accessory mesial (if present) and distal ones strongly curved posteriorly (2); mesiodistally symmetrical postcanines with bulbous main cusp A and accessory ones (3); leaf-shaped postcanines (4); labial sectorial border with lingual platform (5).

122. Number of upper postcanine teeth in adults: (0) no greater than 10; (1) greater than 11.

Postcranial skeleton

123. Vertebral centra: amphicoelous (0); platycoelous (1).

124. Axis centrum: cylindrical (0) or depressed (1).

125. Dens: absent or vestigial (0) or strongly developed (1).

126. Posterior thoracic vertebrate (or middle of the dorsal vertebrate): neural spines slightly inclined or nearly vertical (0) or strongly inclined (1).

127. Anapophysis: absent (0); present (1).

128. Expanded costal plates on dorsal ribs: absent (0); present (1).

129. Lumbar costal plates with ridge overlapping preceding rib: absent (0); present (1).

130. Acromion process: absent (0); weak to moderate (1); strongly developed and close to level of glenoid (2).

131. Scapular constriction below the acromion process: absent (0); present (1).

132. Scapular elongation between the acromion and glenoid: absent (0); present (1).

133. Procoracoid in glenoid: present (0); barely present or absent (1).

134. Procoracoid contact with scapula: greater than coracoid contact (0); equal to or less than coracoid contact (1).

135. Humeral ectepicondylar foramen: present (0); absent (1).

136. Ulnar olecranon process: unossified or poorly ossified (0); well ossified (1).

137. Manual digit III phalanx number: four (0); three (1).

138. Manual digit IV phalanx number: four (0); three (1).

139. Dorsal profile of ilium: strongly convex (0); flat to concave (1).

140. Length of anterior process of ilium anterior to acetabulum (relative to diameter of acetabulum): less than 1.5 (0); greater than 1.5 (1).

141. Lateral surface of iliac blade: concave or nearly flat (0); convex (1); a longitudinal ridge divides it into dorsal and ventral moieties (1).

142. Posterior iliac spine: robust and extends beyond acetabulum (0); reduced to small nub that lies entirely anterior to acetabulum (1).

143. Cotyloid (acetabular) notch: lies between the ischial and iliac part of the acetabulum, mainly on ilium (0); between acetabular facet and pubic process of the ischium, on ischium (1).

144. The diameter of the obturator foramen greater than that of the acetabulum: absent (0); present (1).

145. Femoral head: rounded and predominately in plane of shaft (0); subspherical and inflected dorsally (1).

146. Greater trochanter separated from femoral head by distinct notch: absent (0); present (1).

147. Lesser trochanter position: on ventromedial surface of femoral shaft (0); on medial surface of femoral shaft (1).

148. Lesser trochanter location near the level of the femoral head: absent (0); present (1).

Paleoneurological characters

149. Cochlear canal: absent or shallow (forms a short triangular extension of the vestibule at best) (0), excavates a deep recess that can be easily separated from the rest of the vestibule (1).

150. Maxillary canal external nasal ramus: present (0), reduced or absent (1).

151. Maxillary canal internal nasal and superior labial rami: present (0), reduced or absent (1).

152. Lacrimal duct and zygomaticofacial canal: fused (0), separated (1).

153. Maxillary antrum and maxillary canal: fused (0), separated (1).

154. Anteroposteriorly elongated maxillary antrum: absent (0), present (1).

155. Cranial endocast, unossified zone: presence (0), absence (1).

156. Cranial endocast, pineal body: presence (0), absence (1).

157. Cranial endocast, interhemispheric sulcus dividing the cerebral hemispheres: absence (0), presence (1).

158. Cranial endocast, olfactory bulbs region subequal or slightly wider than the area of the cerebral hemispheres (0), cerebral hemispheres region markedly wider than the region of the olfactory bulbs (1).

6. Supplementary Text – Phylogenetic analysis results

We obtained eight most parsimonious trees of 496 steps, with a consistence index of 0.466 and a retention index of 0.783. The Bremer support [107] (=decay indices) were calculated, and

as a measure of branch stability, a bootstrap resampling analysis [108] was conducted, performing 10,000 pseudoreplications (Figure S1). Both absolute and GC (i.e., difference between the frequency whereby the original group and the most frequent contradictory group are recovered in the pseudoreplications [109]) bootstrap frequencies were reported. The Bayesian analysis recovered a standard deviation of split frequencies of 0.008321 after one million generations, indicating that the two chains had reached convergence. A strict consensus tree was produced (Figure S2) and posterior probabilities were calculated for each branch.

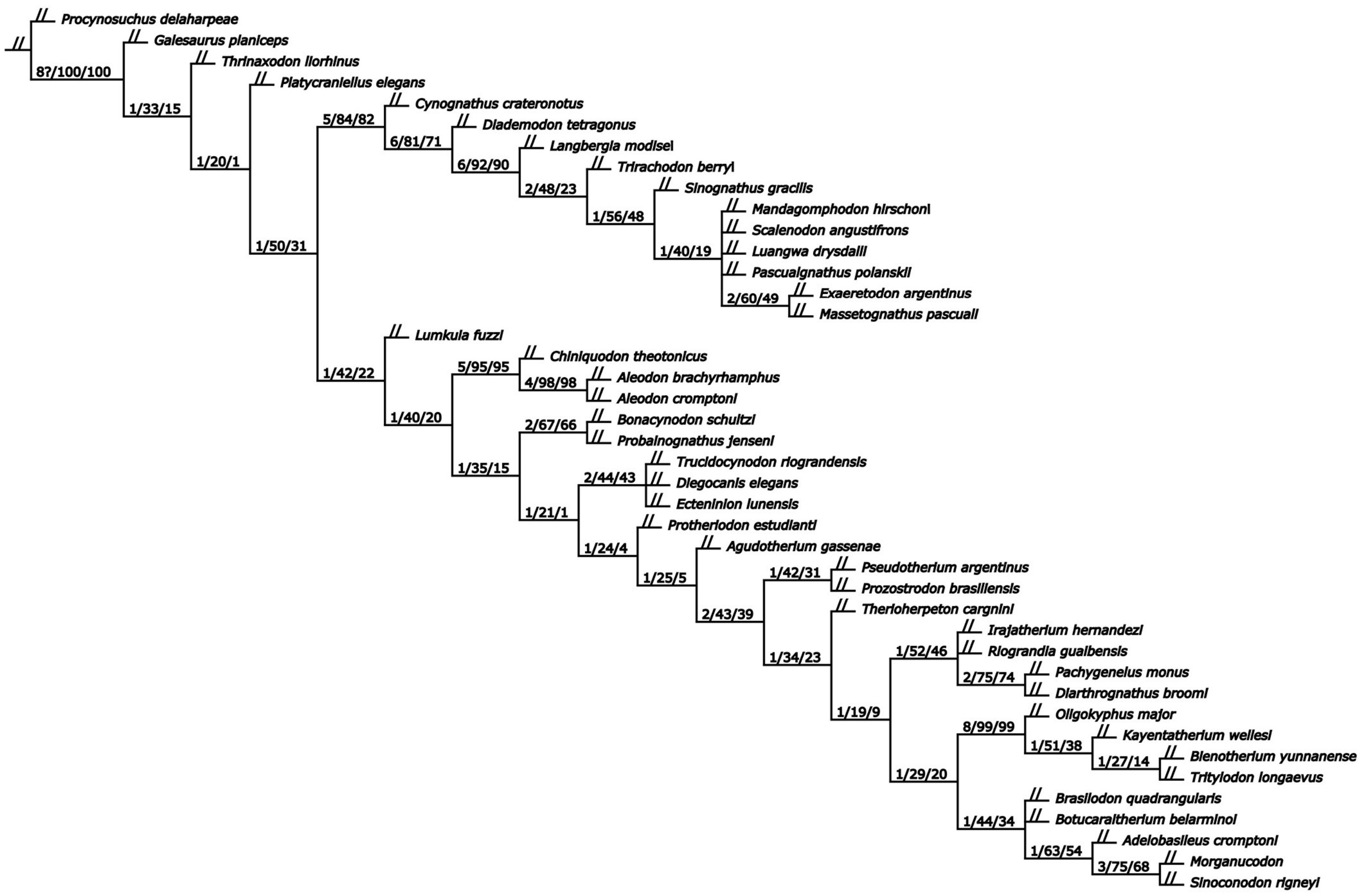

7. Figure S1: Strict consensus tree of cynodont relationships produced by parsimony analysis in this study. Values along each branch denote (from bottom/left to top/right) Bremer support, and absolute and GC (group present/contradicted) bootstrap frequencies for that branch.

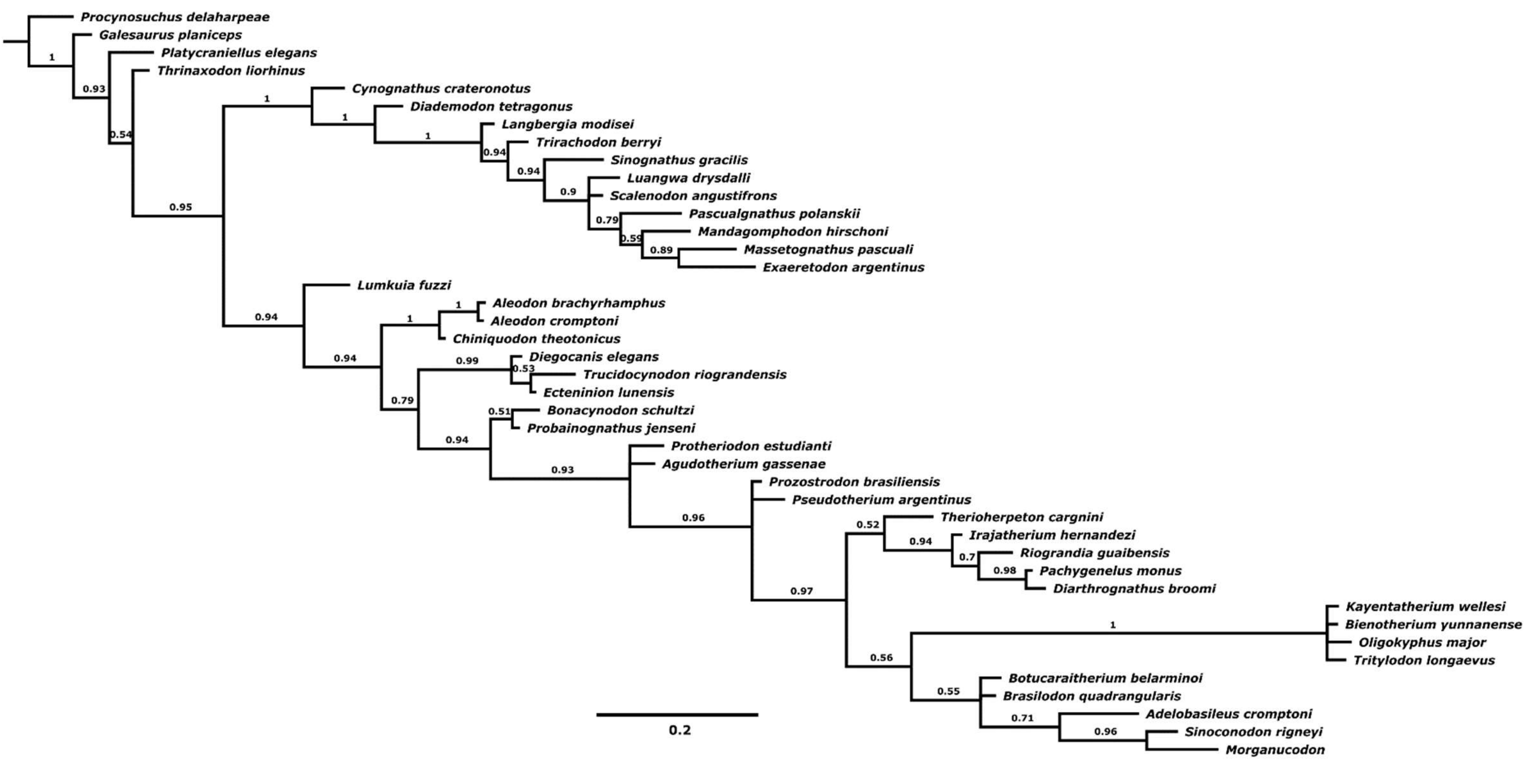

8. Figure S2: Consensus tree of cynodont relationships produced from Bayesian analysis in this study. Posterior probability values are shown along each branch and scale bar denotes scale for branch length. Further details of the analysis can be found in the methods section.

Institution	Specimen no.	Genus	Material	Specimen length (mm)	Postdentary preserved length × height at articular (mm)	First published in	Scanning Details	Voxel Size (mm)	Notes on preservation
UFRGS-PV-T	594	*Brasilodon*	Skull, jaws, isolated postcrania	20.0 (skull and jaws)	5.81 × 1.43	Bonaparte et al., 2003 (formerly holotype of *Brasilitherium riograndensis*)	University of Bristol, Nikon XTH225 ST Micro CT	0.005790	Left jaw in good condition, with incomplete postdentaries and coronoid
UFRGS-PV-T	628	*Brasilodon*	Skull, left jaw	42.7	17.2 × 2.27	Bonaparte et al., 2005	University of Bristol, Nikon XTH225 ST Micro CT	0.015336	Preserves complete skull with little fragmentation, plus lower jaw with good postdentaries
UFRGS-PV-T	628	*Brasilodon*	Right jaw	33.9	7.33 × 1.22 (at posterior most point preserved)	Bonaparte et al., 2005	University of Bristol, Nikon XTH225 ST Micro CT	0.007453	Mostly complete jaw lacking some of articular process and coronoid process. Preserves fragmented anterior postdentary bones
UFRGS-PV-T	760	*Brasilodon*	Skull, jaws, humerus, pectoral girdle	22.0 (skull and jaws)	5.86 × 1.45	Bonaparte et al., 2003	University of Bristol, Nikon XTH225 ST Micro CT	0.006257	Good jaws and postdentaries, skull less well-preserved with lots of fragmentation. Preserves quadrate
UFRGS-PV-T	762	*Brasilodon*	Right lower jaw	13.6			University of Finland, Phoenix High-Resolution X-ray x|s Nanofocus	0.006167	Largely complete but missing dorsal part of coronoid process
UFRGS-PV-T	824	*Brasilodon*	Right lower jaw and postcrania	20.3 (jaw)	Too fragmentary		University of Bristol, Nikon XTH225 ST Micro CT	0.017995	Jaw essentially complete and preserves entire outline. A few fragments of postdentaries
UFRGS-PV-T	929	*Brasilodon*	Skull, jaws and postcrania	26.2 (skull and jaws)	5.97 × 1.44	Bonaparte et al., 2005	University of Finland, Phoenix High-Resolution X-ray x|s Nanofocus	0.013333	Mostly complete skull with in-situ quadrate/quadratojugal. Both dentaries present, right more complete. Fragmented postdentary elements on right side
UFRGS-PV-T	1030	*Brasilodon*	Skull and jaws	25.3	9.81 × 0.87	Bonaparte et al., 2010. (formerly holotype of *Minicynodon maieri*)	University of Bristol, Nikon XTH225 ST Micro CT	0.008020	Preserves whole skull and both lower jaws. Good postdentaries on right side
UFRGS-PV-T	1043	*Brasilodon*	Jaws	34.2	15.6 × 2.92	Bonaparte et al., 2012	University of Finland, Phoenix High-Resolution X-ray x|s Nanofocus	0.016667	Complete jaws with little fragmentation. Majority of postdentaries preserved, partial splenials
UFRGS-PV-T	1043	*Brasilodon*	Skull	39.3		Bonaparte et al., 2012	University of Bristol, Nikon XTH225 ST Micro CT	0.012823	Most complete *Brasilodon* skull with complete right quadrate, zygomatic arches and disarticulated stapes
UFRGS-PV-T	759b	*Brasilodon*	Right lower jaw	16.0			University of Bristol, Nikon XTH225 ST Micro CT	0.009655	Some cracks and missing dorsal coronoid process but otherwise well-preserved
NHMUK-PV-R	7119	*Oligokyphus*	Right jaw	51.4		Kühne (1956)	Natural History Museum, London, Nikon Metrology HMX ST 225	0.015004	Preserves postdentary trough and posterior Meckelian groove, coronoid boss and some of coronoid process
NHMUK-PV-R	7121	*Oligokyphus*	Right jaw	48.9		Kühne (1956)	Natural History Museum, London, Zeiss Xradia Versa 520	0.028387	Preserves anterior of postdentary trough, Meckelian groove and most of symphysis; coronoid process absent.
NHMUK-PV-R	7189	*Oligokyphus*	Left posterior postdentaries	8.18	8.39 × 3.35	Kühne (1956)	Natural History Museum, London, Zeiss Xradia Versa 520	0.011822	Complete articular and posterior surangular and prearticular, all fused; clear contact surface for missing angular
NHMUK-PV-R	7190	*Oligokyphus*	Right posterior postdentaries	5.19	5.25 × 3.66	Kühne (1956)	Natural History Museum, London, Zeiss Xradia Versa 520	0.009082	Posterior fused articular, prearticular and surangular, less anterior preserved than 7189 Angular also absent as in 7189, with very clear contact surface
NHMUK-PV-R	7196	*Oligokyphus*	Right quadrate	4.15		Kühne (1956)	Natural History Museum, London, Zeiss Xradia Versa 520	0.011493	Complete trochlea, some damage to dorsal portion

NHMUK-PV-R	7204	*Oligokyphus*	Right dentary two parts	34.9		Kühne (1956)	Natural History Museum, London, Zeiss Xradia Versa 520	0.026149	Anterior section preserves symphysis and Meckelian groove. Posterior section preserves postdentary trough, partial articular process, and ventral part of coronoid process.
NHMUK-PV-R	7373	*Oligokyphus*	Anterior left jaw	32.2		Kühne (1956)	Natural History Museum, London, Nikon Metrology HMX ST 225	0.015004	Preserves anterior jaw region well including medial side showing Meckelian groove and posterior of symphysial area
UFRGS-PV-T	596	*Riograndia*	Skull and jaws	33.6	5.26 × 3.01	Soares et al., 2011	University of Bristol, Nikon XTH225 ST Micro CT	0.012324	Skull very well-preserved with only minor deformation. Preserves complete jaw, postdentaries, quadrate, quadratojugal and squamosal largely in situ
UFRGS-PV-T	622	*Riograndia*	Left lower jaw	26.6		Soares et al., 2011	Pontifical Catholic University, SkyScan 1173 High Energy Micro-CT	0.021161	Complete jaw with very little damage, no postdentary material
UFRGS-PV-T	623	*Riograndia*	Right lower jaw	34.2		Soares et al., 2011	University of Bristol, Nikon XTH225 ST Micro CT	0.019847	Very well preserved, includes whole coronoid process, no postdentary material
UFRGS-PV-T	624	*Riograndia*	Left lower jaw	32.2	8.05 × 3.45		University of Bristol, Nikon XTH225 ST Micro CT	0.008707	Very good lower jaw and postdentary bones, preserves coronoid bone
UFRGS-PV-T	766	*Riograndia*	Left lower jaw	22.0			Pontifical Catholic University, SkyScan 1173 High Energy Micro-CT	0.019046	Preserves trough and Meckel's groove with good detail
UFRGS-PV-T	793	*Riograndia*	Left lower jaw	25.9			University of Bristol, Nikon XTH225 ST Micro CT	0.007975	Mid-section of large lower jaw including trough and sulcus but misses anterior and coronoid process
UFRGS-PV-T	832	*Riograndia*	Skull and jaws	28.9			University of Bristol, Nikon XTH225 ST Micro CT	0.017429	Skull mostly complete but fragmented in places. Left jaw is well-preserved but misses angular process, right jaw less so
UFRGS-PV-T	833	*Riograndia*	Skull and left jaw	29.3 (jaw)	12.2 × 4.02	Soares et al., 2011	University of Finland, Phoenix High-Resolution X-ray x|s Nanofocus	0.018333	Mostly complete but lots of fragmentation on skull, preserves complete zygomatic arch, very good postdentary preservation
UFRGS PV-T	1319	*Riograndia*	Right lower jaw	35.6	Too fragmentary		University of Bristol, Nikon XTH225 ST Micro CT	0.012177	Complete lower jaw but mediolaterally separated at the tooth row. Some postdentary fragments
MPDC	1B1	*Riograndia*	Left lower jaw	25.0			University of Bristol, Nikon XTH225 ST Micro CT	0.014571	Very good preservation and complete specimen, missing only some fragments of the coronoid process

9. Table S1: *Brasilodon*, *Riograndia* and *Oligokyphus* specimens used in this study. Institutional Abbreviations: UFRGS-PV-T, Universidade Federal do Rio Grande do Sul, Rio Grande do Sul, Porto Alegre, Brazil; NHMUK-PV-R, Natural History Museum, London, UK, MPDC, Museu Padre Daniel Cargnin, Mata, Rio Grande do Sul, Brazil.

Taxon	FAD	LAD
Procynosuchus_delaharpeae	257	252
Galesaurus_planiceps	252	249
Thrinaxodon_liorhinus	252	247
Platycraniellus_elegans	250	249
Cynognathus_crateronotus	247	241
Diademodon_tetragonus	246	241
Trirachodon_berryi	246	244
Sinognathus_gracilis	246	241
Langbergia_modisei	247	246
Pascualgnathus_polanskii	235.8	233
Luangwa_drysdalli	238	233
Massetognathus_pascuali	238	233.7
Exaeretodon_argentinus	231.4	221.4
Scalenodon_angustifrons	242	241
Mandagomphodon_hirschoni	238	237
Chiniquodon_theotonicus	236.1	233.7
Lumkuia_fuzzi	235	233
Probainognathus_jenseni	236.1	233.7
Therioherpeton_cargnini	231.4	221.4
Riograndia_guaibensis	225.6	225.6
Irajatherium_hernandezi	225.6	225.6
Diarthrognathus_broomi	201.3	191.1
Pachygenelus_monus	201.3	191.1
Botucaraitherium_belarminoi	225.6	225.6
Brasilodon_quadrangularis	225.6	225.6
Tritylodon_longaevus	201.3	191.1
Oligokyphus_major	201.3	192.9
Pseudotherium_argentinus	231.4	221.4
Bienotherium_yunnanense	202	190
Kayentatherium_wellesi	193.5	186.5
Adelobasileus_cromptoni	228	224
Sinoconodon_rigneyi	197	190
Morganucodon	208	190
Ectenion_lunensis	231.4	221.4
Diegocanis_elegans	231.4	221.4
Trucidocynodon_riograndensis	231.4	221.4
Prozostrodon_brasiliensis	231.4	221.4
Bonacynodon schultzi	238	233.7
Protheriodon estudianti	238	233.7
Aleodon_cromptoni	238	233.7
Aleodon_brachyrhamphus	238	233
Agudotherium_gassenae	227	221.4

10. Table S2: First and last appearance dates used in time calibration of the phylogenetic tree of cynodonts.

11. Supplementary References

93 Bonaparte, J. F., Soares M. B. & Martinelli A. G. Discoveries in the Late Triassic of Brazil improve knowledge on the origin of mammals. *Historia Natural, Fundación Felix de Azara, Tercera Serie 2* **2**, 5-30 (2012).

94 Bonaparte, J. F., Ferigolo, J. & Ribeiro, A. M. A primitive Late Triassic 'ictidosaur'from Rio Grande do Sul, Brazil. *Palaeontology* **44**, 623-635 (2001).

95 Sues, H-D. First record of the tritylodontid *Oligokyphus* (Synapsida) from the Lower Jurassic of western North America. *Journal of Vertebrate Paleontology* **5,** 328-335 (1985).

96 Fourie, S. The jaw articulation of *Tritylodontoideus maximus*. *South African Journal of Science* **64**, 255-265 (1968).

97 Jasinoski, S. C. & Chinsamy, A. Mandibular histology and growth of the nonmammaliaform cynodont *Tritylodon*. *Journal of Anatomy* **220**, 564-579 (2012).

98 Lucas, S.G. & Luo Z-X. *Adelobasileus* from the Upper Triassic of West Texas: the oldest mammal. *Journal of Vertebrate Paleontology* **13**, 309-334 (1993).

99 Martínez, R. N., May, C. L. & Forster, C. A. A new carnivorous cynodonts from the Ischigualasto Formation (Late Triassic, Argentina), with comments on eucynodont phylogeny. *Journal of Vertebrate Paleontology* **16**, 271–284 (1996).

100 Luo, Z-X., Crompton, A. W. 7 Sun, A-L. 2001. A new mammaliaform from the Early Jurassic and evolution of mammalian characteristics. *Science* **292**, 1535-1540 (2001).

101 Sidor, C. A. & Smith, R. M. H. A new galesaurid (Therapsida: Cynodontia) from the Lower Triassic of South Africa. *Palaeontology* **47**, 535-56 (2004).

102 Gaetano, L.C., Abdala, F., Seoane, F.D. et al. A new cynodont from the Upper Triassic Los Colorados Formation (Argentina, South America) reveals a novel paleobiogeographic context for mammalian ancestors. *Scientific reports* **12**, 6451 (2022).

103 Martinelli, A. G., Soares, M. B., Oliveira, T. V., Rodrigues, P. G. & Schultz, C. L. The Triassic eucynodont *Candelariodon barberenai* revisited and the early diversity of stem prozostrodontians. *Acta Palaeontologica Polonica* **62**, 527-542 (2017). https://doi.org/10.4202/app.00344.2017

104 Martinelli, A. G., Ezcurra, M. D., Fiorelli, L. E., Escobar, J., Hechenleitner, E. M., von Baczko, M. B., Taborda, J. R. A. & Desojo, J. B. A new early-diverging probainognathian cynodont and a revision of the occurrence of cf. *Aleodon* from the Chañares Formation, northwestern Argentina: new clues on the faunistic composition of the latest Middle–?earliest Late Triassic Tarjadia Assemblage Zone. *The Anatomical Record*, DOI: 10.1002/ar.25388 (2024).

105 Kerber, L., Miron, L. R., Budabué, J. M. & Martinelli, A. G. Endocranial anatomy of the early prozostrodonts (Eucynodontia: Probainognathia) and the neurosensory evolution of the mammalian forerunners. *The Anatomical Record* **307,** 1442-1473 (2024).

106 Stefanello, M, Martinelli, A. G., Müller, R. T., Dias-da-Silva, S. & Kerber, L. A complete skull of a stem mammal from the Late Triassic of Brazil illuminates the early evolution of prozostrodonts. *Journal of Mammalian Evolution* **30** 299-317 (2023).

107 Bremer, K. Branch support and tree stability. *Cladistics* **10**, 295-304 (1994).

108 Felsenstein, J. Phylogenies and the comparative method. *The American Naturalist* **125**, 1-15 (1985).

109 Goloboff, P. A., Farris, J. S., Källersjö, M., Oxelman, B., Ramírez, M. & Szumik, C. Improvements to resampling measures of group support. *Cladistics* **1** 324-332 (2003).
